# On the subgroup structure of the hyperoctahedral group in six dimensions

**DOI:** 10.1107/S2053273314007712

**Published:** 2014-07-10

**Authors:** Emilio Zappa, Eric C. Dykeman, Reidun Twarock

**Affiliations:** a Department of Mathematics, University of York, York, UK; b Department of Biology, University of York, York, UK; c York Centre for Complex Systems Analysis, University of York, York, UK

**Keywords:** symmetry, crystallographic representation, icosahedral group, hyperoctahedral group, spectral graph theory

## Abstract

The subgroup structure of the hyperoctahedral group in six dimensions is investigated. In particular, the subgroups isomorphic to the icosahedral group are studied. The orthogonal crystallographic representations of the icosahedral group are classified and their intersections and subgroups analysed, using results from graph theory and their spectra.

## Abstract 2

The subgroup structure of the hyperoctahedral group in six dimensions is studied, with particular attention to the subgroups isomorphic to the icosahedral group. The orthogonal crystallographic representations of the icosahedral group are classified, and their intersections are studied in some detail, using a combinatorial approach which involves results from graph theory and their spectra.

## 1. Introduction   

The discovery of quasicrystals in 1984 ▶ by Shechtman *et al.* has spurred the mathematical and physical community to develop mathematical tools in order to study structures with non­crystallographic symmetry.

Quasicrystals are alloys with five-, eight-, ten- and 12-fold symmetry in their atomic positions (Steurer, 2004 ▶), and therefore they cannot be organized as (periodic) lattices. In crystallographic terms, their symmetry group *G* is noncrystallographic. However, the noncrystallographic symmetry leaves a lattice invariant in higher dimensions, providing an integral representation of *G*. If such a representation is reducible and contains a two- or three-dimensional invariant subspace, then it is referred to as a crystallographic representation, following terminology given by Levitov & Rhyner (1988 ▶). This is the starting point to construct quasicrystals *via* the cut-and-project method described by, among others, Senechal (1995 ▶), or as a model set (Moody, 2000 ▶).

In this paper we are interested in icosahedral symmetry. The icosahedral group 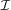 consists of all the rotations that leave a regular icosahedron invariant, it has size 60 and it is the largest of the finite subgroups of 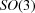. 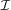 contains elements of order five, therefore it is noncrystallographic in three dimensions; the (minimal) crystallographic representation of it is six-dimensional (Levitov & Rhyner, 1988 ▶). The full icosahedral group, denoted by 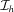, also contains the reflections and is equal to 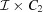, where 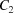 denotes the cyclic group of order two. 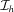 is isomorphic to the Coxeter group 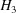 (Humphreys, 1990 ▶) and is made up of 120 elements. In this work, we focus on the icosahedral group *I* because it plays a central role in applications in virology (Indelicato *et al.*, 2011 ▶). However, our considerations apply equally to the larger group 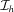.

Levitov & Rhyner (1988 ▶) classified the Bravais lattices in 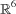 that are left invariant by 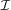: there are, up to equivalence, exactly three lattices, usually referred to as icosahedral Bravais lattices, namely the simple cubic (SC), body-centred cubic (BCC) and face-centred cubic (FCC). The point group of these lattices is the six-dimensional hyperoctahedral group, denoted by 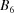, which is a subgroup of 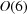 and can be represented in the standard basis of 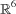 as the set of all 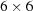 orthogonal and integral matrices. The subgroups of 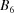 which are isomorphic to the icosahedral group constitute the integral representations of it; among them, the crystallographic ones are those which split, in 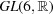, into two three-dimensional irreducible representations of 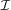. Therefore, they carry two subspaces in 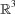 which are invariant under the action of 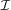 and can be used to model the quasiperiodic structures.

The embedding of the icosahedral group into 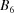 has been used extensively in the crystallographic literature. Katz (1989 ▶), Senechal (1995 ▶), Kramer & Zeidler (1989 ▶), Baake & Grimm (2013 ▶), among others, start from a six-dimensional crystallographic representation of 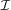 to construct three-dimensional Penrose tilings and icosahedral quasicrystals. Kramer (1987 ▶) and Indelicato *et al.* (2011 ▶) also apply it to study structural transitions in quasicrystals. In particular, Kramer considers in 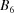 a representation of 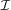 and a representation of the octahedral group 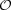 which share a tetrahedral subgroup, and defines a continuous rotation (called Schur rotation) between cubic and icosahedral symmetry which preserves intermediate tetrahedral symmetry. Indelicato *et al*. define a transition between two icosahedral lattices as a continuous path connecting the two lattice bases keeping some symmetry preserved, described by a maximal subgroup of the icosahedral group. The rationale behind this approach is that the two corresponding lattice groups share a common subgroup. These two approaches are shown to be related (Indelicato *et al.*, 2012 ▶), hence the idea is that it is possible to study the transitions between icosahedral quasicrystals by considering two distinct crystallographic representations of 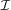 in 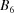 which share a common subgroup.

These papers motivate the idea of studying in some detail the subgroup structure of 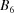. In particular, we focus on the subgroups isomorphic to the icosahedral group and its subgroups. Since the group is quite large (it has 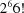 elements), we use for computations the software *GAP* (The GAP Group, 2013 ▶), which is designed to compute properties of finite groups. More precisely, based on Baake (1984 ▶), we generate the elements of 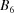 in *GAP* as a subgroup of the symmetric group 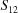 and then find the classes of subgroups isomorphic to the icosahedral group. Among them we isolate, using results from character theory, the class of crystallographic representations of 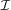. In order to study the subgroup structure of this class, we propose a method using graph theory and their spectra. In particular, we treat the class of crystallographic representations of 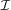 as a graph: we fix a subgroup 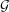 of 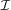 and say that two elements in the class are adjacent if their intersection is equal to a subgroup isomorphic to 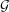. We call the resulting graph 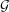-graph. These graphs are quite large and difficult to visualize; however, by analysing their spectra (Cvetkovic *et al.*, 1995 ▶) we can study in some detail their topology, hence describing the intersection and the subgroups shared by different representations.

The paper is organized as follows. After recalling, in §2, the definitions of point group and lattice group, we define, in §3, the crystallographic representations of the icosahedral group and the icosahedral lattices in six dimensions. We provide, following Kramer & Haase (1989 ▶), a method for the construction of the projection into three dimensions using tools from the representation theory of finite groups. In §4 we classify, with the help of *GAP*, the crystallographic representations of 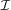. In §5 we study their subgroup structure, introducing the concept of 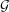-graph, where 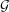 is a subgroup of 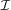.

## 2. Lattices and noncrystallographic groups   

Let 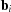, 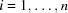 be a basis of 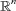, and let 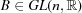 be the matrix whose columns are the components of 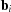 with respect to the canonical basis 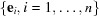 of 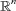. A lattice in 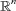 is a 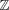-free module of rank *n* with basis *B*, *i.e.*

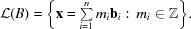
Any other lattice basis is given by *BM*, where 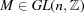, the set of invertible matrices with integral entries (whose determinant is equal to 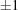) (Artin, 1991 ▶).

The point group of a lattice 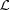 is given by all the orthogonal transformations that leave the lattice invariant (Pitteri & Zanzotto, 2002 ▶): 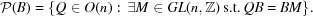



We notice that, if 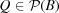, then 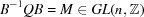. In other words, the point group consists of all the orthogonal matrices which can be represented in the basis *B* as integral matrices. The set of all these matrices constitute the lattice group of the lattice: 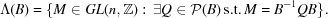



The lattice group provides an integral representation of the point group and these are related *via* the equation 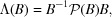
and moreover the following hold (Pitteri & Zanzotto, 2002 ▶): 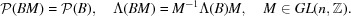



We notice that a change of basis in the lattice leaves the point group invariant, whereas the corresponding lattice groups are conjugated in 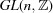. Two lattices are inequivalent if the corresponding lattice groups are not conjugated in 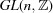 (Pitteri & Zanzotto, 2002 ▶).

As a consequence of the crystallographic restriction [see, for example, Baake & Grimm (2013 ▶)] five- and *n*-fold symmetries, where *n* is a natural number greater than six, are forbidden in dimensions two and three, and therefore any group *G* containing elements of such orders cannot be the point group of a two- or three-dimensional lattice. We therefore call these groups noncrystallographic. In particular, three-dimensional icosahedral lattices cannot exist. However, a noncrystallographic group leaves some lattices invariant in higher dimensions and the smallest such dimension is called the minimal embedding dimension. Following Levitov & Rhyner (1988 ▶), we introduce:

**Definition 2.1**

Let *G* be a noncrystallographic group. A crystallographic representation ρ of *G* is a *D*-dimensional representation of *G* such that:

(1) the characters 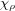 of ρ are integers;

(2) ρ is reducible and contains a two- or three-dimensional representation of *G*.

We observe that the first condition implies that *G* must be the subgroup of the point group of a *D*-dimensional lattice. The second condition tells us that ρ contains either a two- or three-dimensional invariant subspace *E* of 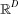, usually referred to as physical space (Levitov & Rhyner, 1988 ▶).

## 3. Six-dimensional icosahedral lattices   

The icosahedral group 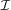 is generated by two elements, 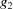 and 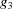, such that 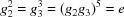, where *e* denotes the identity element. It has size 60 and it is isomorphic to 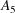, the alternating group of order five (Artin, 1991 ▶). Its character table is given in Table 1 ▶.

From the character table we see that the (minimal) crystallographic representation of 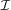 is six-dimensional and is given by 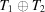. Therefore, 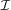 leaves a lattice in 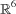 invariant. Levitov & Rhyner (1988 ▶) proved that the three inequivalent Bravais lattices of this type, mentioned in the *Introduction* [Sec sec1] and referred to as icosahedral (Bravais) lattices, are given by, respectively: 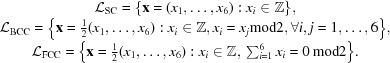



We note that a basis of the SC lattice is the canonical basis of 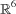. Its point group is given by 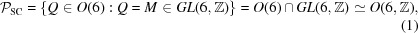
which is the hyperoctahedral group in dimension six. In the following, we will denote this group by 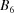, following Humphreys (1996 ▶). We point out that this notation comes from Lie theory: indeed, 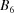 represents the root system of the Lie algebra 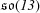 (Fulton & Harris, 1991 ▶). However, the corresponding reflection group 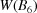 is isomorphic to the hyperoctahedral group in six dimensions (Humphreys, 1990 ▶).

All three lattices have point group 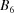, whereas their lattice groups are different and, indeed, they are not conjugate in 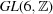 (Levitov & Rhyner, 1988 ▶).

Let 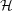 be a subgroup of 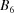 isomorphic to 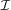. 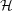 provides a (faithful) integral and orthogonal representation of 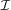. Moreover, if 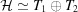 in 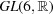, then 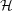 is also crystallographic (in the sense of Definition 2.1[Statement definition2.1]). All of the other crystallographic representations are given by 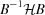, where 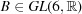 is a basis of an icosahedral lattice in 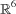. Therefore we can focus our attention, without loss of generality, on the orthogonal crystallographic representations.

### 3.1. Projection operators   

Let 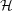 be a crystallographic representation of the icosahedral group. 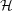 splits into two three-dimensional irreducible representations (IRs), 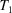 and 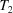, in 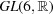. This means that there exists a matrix 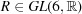 such that 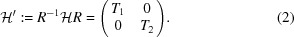



The two IRs 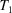 and 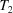 leave two three-dimensional subspaces invariant, which are usually referred to as the physical (or parallel) space 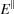 and the orthogonal space 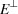 (Katz, 1989 ▶). In order to find the matrix *R* (which is not unique in general), we follow (Kramer & Haase, 1989 ▶) and use results from the representation theory of finite groups (for proofs and further results see, for example, Fulton & Harris, 1991 ▶). In particular, let 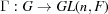 be an *n*-dimensional representation of a finite group *G* over a field *F* (*F* = 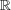, 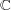). By Maschke’s theorem, Γ splits, in 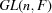, as 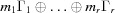, where 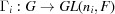 is an 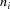-dimensional IR of *G*. Then the projection operator 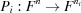 is given by 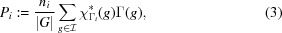
where 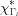 denotes the complex conjugate of the character of the representation 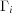. This operator is such that its image 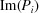 is equal to an 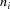-dimensional subspace 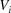 of 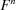 invariant under 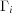. In our case, we have two projection operators, 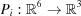, *i* = 1, 2, corresponding to the IRs 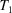 and 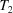, respectively. We assume the image of 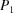, 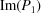, to be equal to 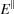, and 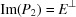. If 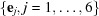 is the canonical basis of 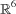, then a basis of 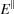 (respectively 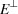) can be found considering the set 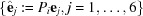 for 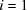 (respectively 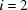) and then extracting a basis 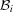 from it. Since 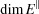 = 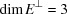, we obtain 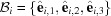, for *i* = 1, 2. The matrix *R* can be thus written as 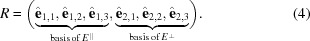



Denoting by 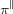 and 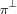 the 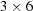 matrices which represent 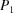 and 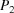 in the bases 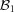 and 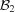, respectively, we have, by linear algebra 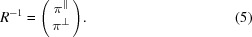



Since 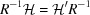 [*cf.* equation (2)], we obtain 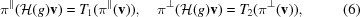
for all 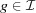 and 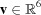. In particular, the following diagram commutes
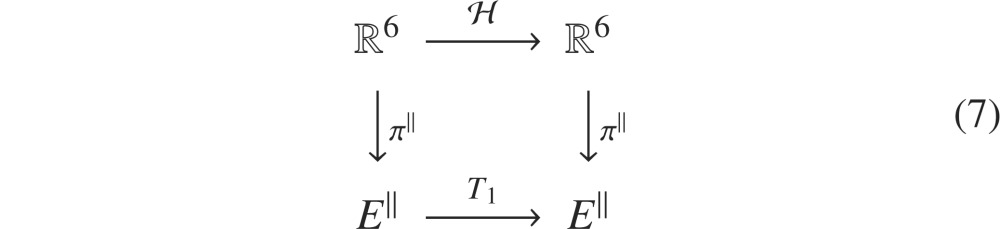


The set 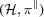 is the starting point for the construction of quasicrystals *via* the cut-and-project method (Senechal, 1995 ▶; Indelicato *et al.*, 2012 ▶).

## Crystallographic representations of 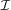   

4.

From the previous section it follows that the six-dimensional hyperoctahedral group 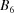 contains all the (minimal) orthogonal crystallographic representations of the icosahedral group. In this section we classify them, with the help of the computer software programme *GAP* (The GAP Group, 2013 ▶).

### Representations of the hyperoctahedral group 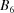   

4.1.

Permutation representations of the *n*-dimensional hyperoctahedral group 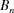 in terms of elements of 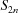, the symmetric group of order 2*n*, have been described by Baake (1984 ▶). In this subsection, we review these results because they allow us to generate 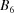 in *GAP* and further study its subgroup structure.

It follows from equation (1) that 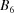 consists of all the orthogonal integral matrices. A matrix 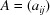 of this kind must satisfy 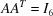, the identity matrix of order six, and have integral entries only. It is easy to see that these conditions imply that *A* has entries in {0, ±1} and each row and column contains 1 or −1 only once. These matrices are called signed permutation matrices. It is straightforward to see that any 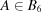 can be written in the form *NQ*, where *Q* is a 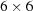 permutation matrix and *N* is a diagonal matrix with each diagonal entry being either 1 or −1. We can thus associate with each matrix in 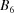 a pair 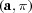, where 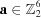 is a vector given by the diagonal elements of *N*, and 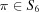 is the permutation associated with *Q*. The set of all these pairs constitutes a group (called the wreath product of 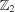 and 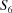, and denoted by 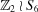; Humphreys, 1996 ▶) with the multiplication rule given by 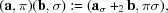
where 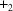 denotes addition modulo 2 and 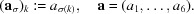


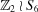
 and 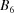 are isomorphic, an isomorphism *T* being the following: 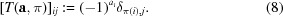



It immediately follows that |*B*_6_| = 2^6^6! = 46 080. A set of generators is given by 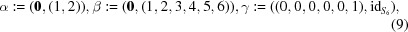
which satisfy the relations 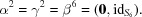



Finally, the function 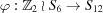 defined by 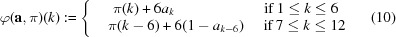
is injective and maps any element of 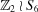 into a permutation of 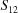, and provides a faithful permutation representation of 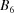 as a subgroup of 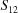. Combining equation (8) with the inverse of equation (10) we get the function 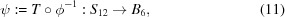
which can be used to map a permutation into an element of 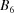.

### 4.2. Classification   

**Figure 1:**
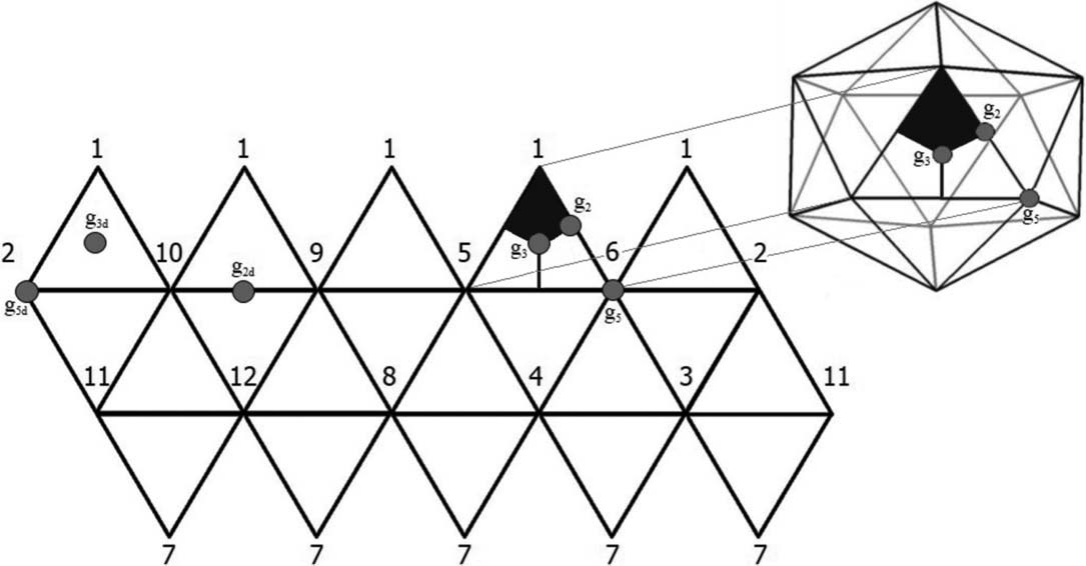
A planar representation of an icosahedral surface, showing our labelling convention for the vertices; the dots represent the locations of the symmetry axes corresponding to the generators of the icosahedral group and its subgroups. The kite highlighted is a fundamental domain of the icosahedral group.

In this subsection we classify the orthogonal crystallographic representations of the icosahedral group. We start by recalling a standard way to construct such a representation, following Zappa *et al.* (2013 ▶). We consider a regular icosahedron and we label each vertex by a number from one to 12, so that the vertex opposite to vertex *i* is labelled by 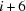 (see Fig. 1 ▶). This labelling induces a permutation representation 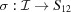 given by 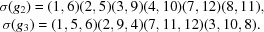
Using equation (11) we obtain a representation 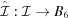 given by 
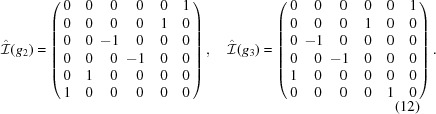



We see that 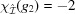 and 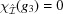, so that, by looking at the character table of 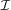, we have 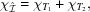
which implies, using Maschke’s theorem (Fulton & Harris, 1991 ▶), that 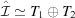 in 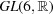. Therefore, the subgroup 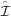 of 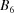 is a crystallographic representation of 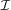.

Before we continue, we recall the following (Humphreys, 1996 ▶):

**Definition 4.1**

Let *H* be a subgroup of a group *G*. The conjugacy class of *H* in *G* is the set 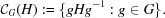

In order to find all the other crystallographic representations, we use the following scheme:

(*a*) we generate 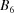 as a subgroup of 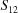 using equations (9) and (10);

(*b*) we list all the conjugacy classes of the subgroups of 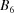 and find a representative for each class;

(*c*) we isolate the classes whose representatives have order 60;

(*d*) we check if these representatives are isomorphic to 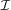;

(*e*) we map these subgroups of 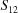 into 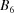 using equation (11) and isolate the crystallographic ones by checking the characters; denoting by *S* the representative, we decompose 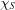 as 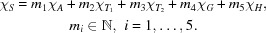



Note that *S* is crystallographic if and only if 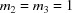 and 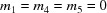.

We implemented steps (1)–(4) in *GAP* (see Appendix C[App appc]). There are three conjugacy classes of subgroups isomorphic to 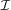 in 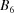. Denoting by 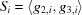 the representatives of the classes returned by *GAP*, we have, using equation (11), 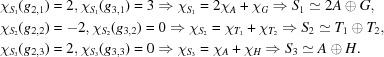



Since 2*A* is decomposable into two one-dimensional representations, it is not strictly speaking two dimensional in the sense of Definition 2.1[Statement definition2.1], and as a consequence, only the second class contains the crystallographic representations of 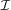. A computation in *GAP* shows that its size is 192. We thus have the following:

**Proposition 4.1**

The crystallographic representations of 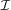 in 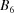 form a unique conjugacy class in the set of all the classes of subgroups of 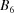, and its size is equal to 192.

We briefly point out that the other two classes of subgroups isomorphic to 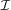 in 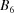 have an interesting algebraic intepretation. First of all, we observe that 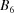 is an extension of 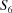, since according to Humphreys (1996 ▶): 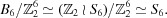



Following Janusz & Rotman (1982 ▶), it is possible to embed the symmetric group 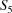 into 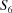 in two different ways. The canonical embedding is achieved by fixing a point in 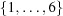 and permuting the other five, whereas the other embedding is by means of the so-called ‘exotic map’ 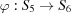, which acts on the six 5-Sylow subgroups of 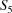 by conjugation. Recalling that the icosahedral group is isomorphic to the alternating group 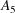, which is a normal subgroup of 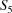, then the canonical embedding corresponds to the representation 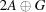 in 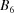, while the exotic one corresponds to the representation 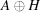.

In what follows, we will consider the subgroup 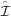 previously defined as a representative of the class of the crystallographic representations of 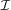, and denote this class by 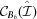.

Recalling that two representations 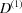 and 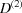 of a group *G* are said to be equivalent if there are related *via* a similarity transformation, *i.e.* there exists an invertible matrix *S* such that 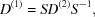
then an immediate consequence of Proposition 4.1[Statement proposition4.1] is the following:

**Corollary 4.1**

Let 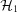 and 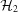 be two orthogonal crystallographic representations of 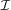. Then 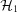 and 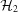 are equivalent in 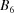.

We observe that the determinant of the generators of 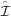 in equation (12) is equal to one, so that 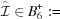 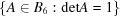. Proposition 4.1[Statement proposition4.1] implies that all the crystallographic representations belong to 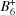. The remarkable fact is that they split into two different classes in 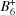. To see this, we first need to generate 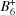. In particular, with *GAP* we isolate the subgroups of index two in 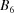, which are normal in 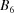, and then, using equation (11), we find the one whose generators have determinant equal to one. In particular, we have 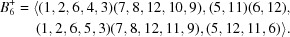



We can then apply the same procedure to find the crystallographic representations of 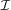, and see that they split into two classes, each one of size 96. Again we can choose 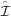 as a representative for one of these classes; a representative 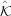 for the other one is given by 
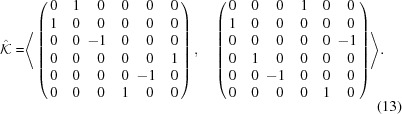



We note that in the more general case of 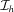, we can construct the crystallographic representations of 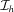 starting from the crystallographic representations of 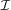. First of all, we recall that 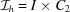, where 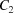 is the cyclic group of order two. Let 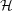 be a crystallographic representation of 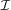 in 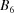, and let 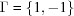 be a one-dimensional representation of 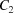. Then the representation 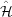 given by 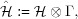
where 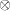 denotes the tensor product of matrices, is a representation of 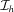 in 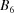 and it is crystallographic in the sense of Definition 2.1[Statement definition2.1] (Fulton & Harris, 1991 ▶).

### 4.3. Projection into the three-dimensional space   

We study in detail the projection into the physical space 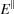 using the methods described in §3.1.

Let 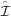 be the crystallographic representation of 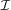 given in equation (12). Using equation (3) with 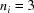 and 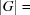 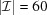 we obtain the following projection operators 
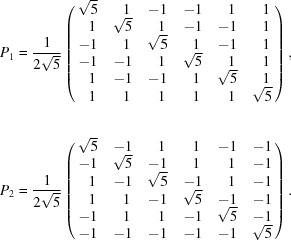
The rank of these operators is equal to three. We choose as a basis of 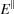 and 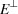 the following linear combination of the columns 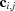 of the projection operators 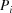, for *i * = 1, 2 and 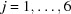: 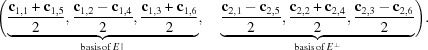



With a suitable rescaling, we obtain the matrix *R* given by 
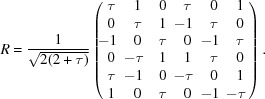



The matrix *R* is orthogonal and reduces 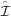 as in equation (2). In Table 2 ▶ we give the explicit forms of the reduced representation. The matrix representation in 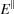 of 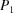 is given by [see equation (5)] 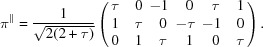



The orbit 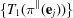, where 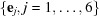 is the canonical basis of 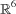, represents a regular icosahedron in three dimensions centred at the origin (Senechal, 1995 ▶; Katz, 1989 ▶; Indelicato *et al.*, 2011 ▶).

Let 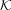 be another crystallographic representation of 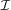 in 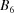. By Proposition 4.1[Statement proposition4.1], 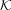 and 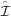 are conjugated in 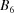. Consider 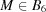 such that 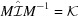 and let 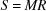. We have 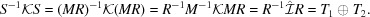
Therefore it is possible, with a suitable choice of the reducing matrices, to project all the crystallographic representations of 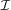 in 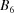 in the same physical space.

## 5. Subgroup structure   

The nontrivial subgroups of 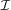 are listed in Table 3 ▶, together with their generators (Hoyle, 2004 ▶). Note that 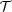, 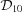 and 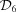 are maximal subgroups of 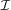, and that 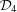, 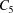 and 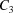 are normal subgroups of 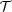, 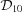 and 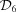, respectively (Humphreys, 1996 ▶; Artin, 1991 ▶). The permutation representations of the generators in 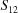 are given in Table 4 ▶ (see also Fig. 1 ▶).

Since 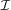 is a small group, its subgroup structure can be easily obtained in *GAP* by computing explicitly all its conjugacy classes of subgroups. In particular, there are seven classes of nontrivial subgroups in 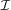: any subgroup *H* of 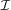 has the property that, if *K* is another subgroup of 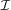 isomorphic to *H*, then *H* and *K* are conjugate in 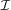 (this property is referred to as the ‘friendliness’ of the subgroup *H*; Soicher, 2006 ▶). In other words, denoting by 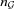 the number of subgroups of 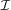 isomorphic to 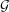, *i.e.*

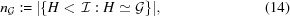
we have (*cf.* Definition 4.1[Statement definition4.1])
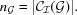



In Table 5 ▶ we list the size of each class of subgroups in 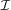. Geometrically, different copies of 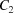, 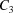 and 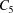 correspond to the different two-, three- and fivefold axes of the icosahedron, respectively. In particular, different copies of 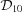 stabilize one of the six fivefold axes of the icosahedron, and each copy of 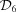 stabilizes one of the ten threefold axes. Moreover, it is possible to inscribe five tetrahedra into a dodecahedron, and each different copy of the tetrahedral group in 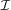 stabilizes one of these tetrahedra.

### Subgroups of the crystallographic representations of 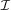   

5.1.

Let 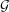 be a subgroup of 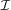. The function (11) provides a representation of 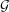 in 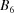, denoted by 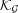, which is a subgroup of 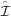. Let us denote by 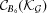 the conjugacy class of 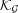 in 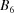. The next lemma shows that this class contains all the subgroups of the crystallographic representations of 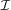 in 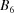.

**Lemma 5.1**

Let 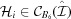 be a crystallographic representation of 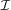 in 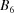 and let 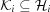 be a subgroup of 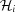 isomorphic to 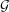. Then 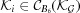.

**Proof**

Since 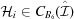, there exists 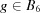 such that 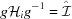, and therefore 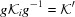 is a subgroup of 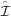 isomorphic to 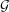. Since all these subgroups are conjugate in 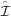 [they are ‘friendly’ in the sense intended by Soicher (2006 ▶)], there exists 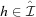 such that 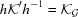. Thus 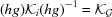, implying that 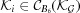.

□

We next show that every element of 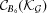 is a subgroup of a crystallographic representation of 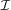.

**Lemma 5.2**

Let 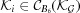. There exists 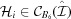 such that 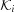 is a subgroup of 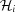.

**Proof**

Since 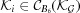, there exists 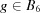 such that 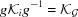. We define 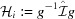. It can be seen immediately that 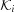 is a subgroup of 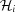.

□

As a consequence of these lemmata, 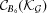 contains all the subgroups of 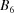 which are isomorphic to 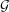 and are subgroups of a crystallographic representation of 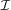. The explicit forms of 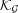 are given in Appendix B[App appb]. We point out that it is possible to find subgroups of 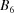 isomorphic to a subgroup 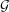 of 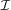 which are not subgroups of any crystallographic representation of 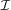. For example, the following subgroup 
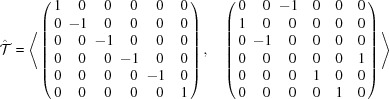
is isomorphic to the tetrahedral group 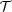; a computation in *GAP* shows that it is not a subgroup of any elements in 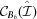. Indeed, the two classes of subgroups, 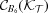 and 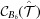, are disjoint.

Using *GAP*, we compute the size of each 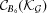 (see Table 5 ▶). We observe that 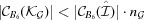. This implies that crystallographic representations of 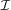 may share subgroups. In order to describe more precisely the subgroup structure of 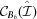 we will use some basic results from graph theory and their spectra, which we are going to recall in the next section[Sec sec5.2].

### 5.2. Some basic results of graph theory and their spectra   

In this section we recall, without proofs, some concepts and results from graph theory and spectral graph theory. Proofs and further results can be found, for example, in Foulds (1992 ▶) and Cvetkovic *et al.* (1995 ▶).

Let *G* be a graph with vertex set 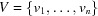. The number of edges incident with a vertex *v* is called the degree of *v*. If all vertices have the same degree *d*, then the graph is called regular of degree *d*. A walk of length *l* is a sequence of *l* consecutive edges and it is called a path if they are all distinct. A circuit is a path starting and ending at the same vertex and the girth of the graph is the length of the shortest circuit. Two vertices *p* and *q* are connected if there exists a path containing *p* and *q*. The connected component of a vertex *v* is the set of all vertices connected to *v*.

The adjacency matrix *A* of *G* is the 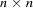 matrix 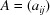 whose entries 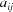 are equal to one if the vertex 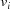 is adjacent to the vertex 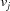, and zero otherwise. It can be seen immediately from its definition that *A* is symmetric and 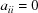 for all *i*, so that 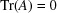. It follows that *A* is diagonalisable and all its eigenvalues are real. The spectrum of the graph is the set of all the eigenvalues of its adjacency matrix *A*, usually denoted by 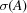.

**Theorem 5.1**

Let *A* be the adjacency matrix of a graph *G* with vertex set 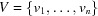. Let 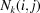 denote the number of walks of length *k* starting at vertex 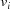 and finishing at vertex 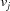. We have 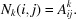

We recall that the spectral radius of a matrix *A* is defined by 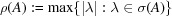. If *A* is a non-negative matrix, *i.e.* if all its entries are non-negative, then 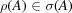 (Horn & Johnson, 1985 ▶). Since the adjacency matrix of a graph is non-negative, 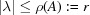, where 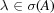 and *r* is the largest eigenvalue. *r* is called the index of the graph *G*.

**Theorem 5.2**

Let 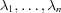 be the spectrum of a graph *G*, and let *r* denote its index. Then *G* is regular of degree *r* if and only if 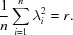
Moreover, if *G* is regular the multiplicity of its index is equal to the number of its connected components.

### 5.3. Applications to the subgroup structure   

Let 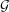 be a subgroup of 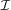. In the following we represent the subgroup structure of the class of crystallographic representations of 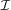 in 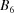, 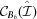, as a graph. We say that 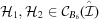 are adjacent to each other (*i.e.* connected by an edge) in the graph if there exists 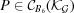 such that 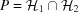. We can therefore consider the graph 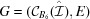, where an edge 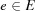 is of the form 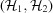. We call this graph 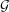-graph.

Using *GAP*, we compute the adjacency matrices of the 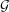-graphs. The algorithms used are shown in Appendix C[App appc]. The spectra of the 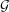-graphs are given in Table 6 ▶. We first of all notice that the adjacency matrix of the 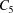-graph is the null matrix, implying that there are no two representations sharing precisely a subgroup isomorphic to 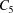, *i.e.* not a subgroup containing 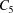. We point out that, since the adjacency matrix of the 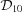-graph is not the null one, then there exist crystallographic representations, say 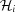 and 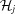, sharing a maximal subgroup isomorphic to 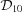. Since 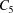 is a (normal) subgroup of 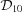, then 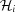 and 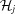 do share a 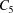 subgroup, but also a 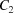 subgroup. In other words, if two representations share a fivefold axis, then necessarily they also share a twofold axis.

A straightforward calculation based on Theorem 5.2[Statement theorem5.2] leads to the following

**Proposition 5.1**

Let 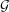 be a subgroup of 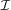. Then the corresponding 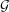-graph is regular.

In particular, the degree 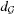 of each 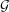-graph is equal to the largest eigenvalue of the corresponding spectrum. As a consequence we have the following:

**Proposition 5.2**

Let 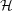 be a crystallographic representation of 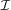 in 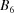. Then there are exactly 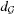 representations 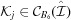 such that 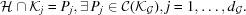
In particular, we have 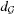 = 5, 6, 10, 0, 30, 20, 60 and 60 for 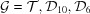, 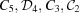 and 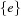, respectively.

In particular, this means that for any crystallographic representation of 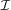 there are precisely 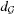 other such representations which share a subgroup isomorphic to 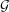. In other words, we can associate to the class 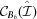 the ‘subgroup matrix’ *S* whose entries are defined by 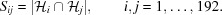



The matrix *S* is symmetric and 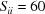, for all *i*, since the order of 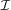 is 60. It follows from Proposition 5.2[Statement proposition5.2] that each row of *S* contains 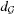 entries equal to 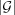. Moreover, a rearrangement of the columns of *S* shows that the 192 crystallographic representations of 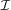 can be grouped into 12 sets of 16 such that any two of these representations in such a set of 16 share a 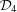-subgroup. This implies that the corresponding subgraph of the 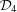-graph is a complete graph, *i.e.* every two distinct vertices are connected by an edge. From a geometric point of view, these 16 representations correspond to ‘six-dimensional icosahedra’. This ensemble of 16 such icosahedra embedded into a six-dimensional hypercube can be viewed as a six-dimensional analogue of the three-dimensional ensemble of five tetrahedra inscribed into a dodecahedron, sharing pairwise a 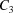-subgroup.

We notice that, using Theorem 5.2[Statement theorem5.2], not all the graphs are connected. In particular, the 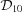
- and the 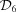-graphs are made up of six connected components, whereas the 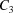
- and the 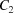-graphs consist of two connected components. With *GAP*, we implemented a breadth-first search algorithm (Foulds, 1992 ▶), which starts from a vertex *i* and then ‘scans’ for all the vertices connected to it, which allows us to find the connected components of a given 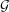-graph (see Appendix C[App appc]). We find that each connected component of the 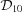
- and 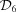-graphs is made up of 32 vertices, while for the 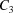
- and 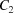-graphs each component consists of 96 vertices. For all other subgroups, the corresponding 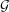-graph is connected and the connected component contains trivially 192 vertices.

We now consider in more detail the case when 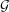 is a maximal subgroup of 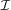. Let 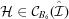 and let us consider its vertex star in the corresponding 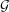-graph, *i.e.*

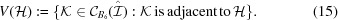



A comparison of Tables 5 ▶ and 6 ▶ shows that 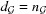 [*i.e.* the number of subgroups isomorphic to 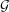 in 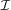, *cf.* equation (14)] and therefore, since the graph is regular, 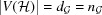. This suggests that there is a one-to-one correspondence between elements of the vertex star of 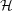 and subgroups of 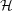 isomorphic to 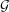; in other words, if we fix any subgroup *P* of 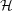 isomorphic to 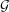, then *P* ‘connects’ 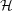 with exactly another representation 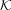. We thus have the following:

**Proposition 5.3**

Let 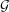 be a maximal subgroup of 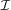. Then for every 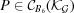 there exist exactly two crystallographic representations of 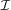, 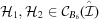, such that 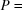 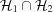.

In order to prove it, we first need the following lemma:

**Lemma 5.3**

Let 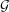 be a maximal subgroup of 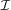. Then the corresponding 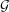-graph is triangle-free, *i.e.* it has no circuits of length three.

**Proof**

Let 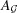 be the adjacency matrix of the 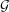-graph. By Theorem 5.1[Statement theorem5.1], its third power 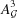 determines the number of walks of length three, and in particular its diagonal entries, 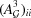, for 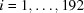, correspond to the number of triangular circuits starting and ending in vertex *i*. A direct computation shows that 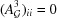, for all *i*, thus implying the non-existence of triangular circuits in the graph.

□

**Proof of Proposition 5.3[Statement proposition5.3]**

If 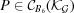, then, using Lemma 5.2[Statement lemma5.2], there exists 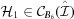 such that *P* is a subgroup of 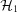. Let us consider the vertex star 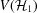. We have 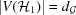; we call its elements 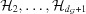. Let us suppose that *P* is not a subgroup of any 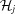, for 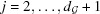. This implies that *P* does not connect 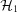 with any of these 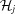. However, since 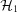 has exactly 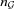 different subgroups isomorphic to 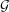, then at least two vertices in the vertex star, say 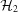 and 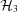, are connected by the same subgroup isomorphic to 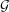, which we denote by *Q*. Therefore we have
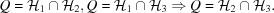
This implies that 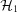, 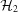 and 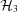 form a triangular circuit in the graph, which is a contradiction due to Lemma 5.3[Statement lemma5.3], hence the result is proved.

□

It is noteworthy that the situation in 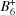 is different. If we denote by 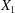 and 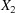 the two disjoint classes of crystallographic representations of 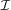 in 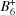 [*cf.* equation (13)], we can build, in the same way as described before, the 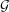-graphs for 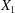 and 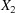, for 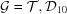 and 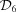. The result is that the adjacency matrices of all these six graphs are the null matrix of dimension 96. This implies that these graphs have no edges, and so the representations in each class do not share any maximal subgroup of 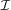. As a consequence, we have the following:

**Proposition 5.4**

Let 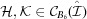 be two crystallographic representations of 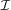, and 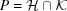, 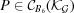, where 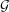 is a maximal subgroup of 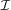. Then 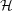 and 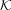 are not conjugated in 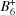. In other words, the elements of 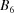 which conjugate 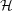 with 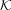 are matrices with determinant equal to −1.

We conclude by showing a computational method which combines the result of Propositions 4.1[Statement proposition4.1] and 5.2[Statement proposition5.2]. We first recall the following:

**Definition 5.1**

Let *H* be a subgroup of a group *G*. The normaliser of *H* in *G* is given by 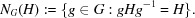

**Corollary 5.1**

Let 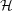 and 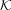 be two crystallographic representations of 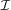 in 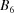 and 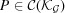 such that 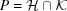. Let 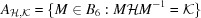
be the set of all the elements of 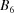 which conjugate 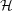 with 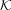 and let 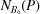 be the normaliser of *P* in 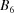. We have 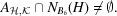
In other words, it is possible to find a nontrivial element 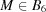 in the normaliser of *P* in 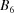 which conjugates 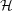 with 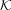.

**Proof**

Let us suppose 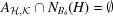. Then 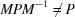, for all 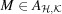. This implies, since 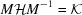, that *P* is not a subgroup of 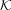, which is a contradiction.

□

We give now an explicit example. We consider the representation 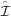 as in equation (12), and its subgroup 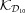 (the explicit form is given in Appendix B[App appb]). With *GAP*, we find the other representation 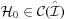 such that 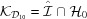. Its explicit form is given by 
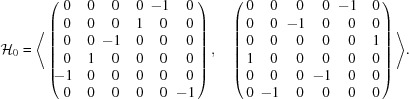



A direct computation shows that the matrix 
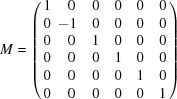
belongs to 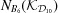 and conjugate 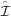 with 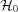. Note that 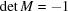.

## 6. Conclusions   

In this work we explored the subgroup structure of the hyperoctahedral group in six dimensions. In particular we found the class of the crystallographic representations of the icosahedral group, whose size is 192. Any such representation, together with its corresponding projection operator 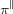, can be chosen to construct icosahedral quasicrystals *via* the cut-and-project method. We then studied in detail the subgroup structure of this class. For this, we proposed a method based on spectral graph theory and introduced the concept of 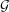-graph, for a subgroup 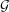 of the icosahedral group. This allowed us to study the intersection and the subgroups shared by different representations. We have shown that, if we fix any representation 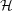 in the class and a maximal subgroup *P* of 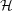, then there exists exactly one other representation 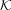 in the class such that 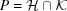. As explained in the *Introduction* [Sec sec1], this can be used to describe transitions which keep intermediate symmetry encoded by *P*. In particular, this result implies in this context that a transition from a structure arising from 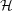 *via* projection will result in a structure obtainable for 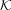 *via* projection if the transition has intermediate symmetry described by *P*. Therefore, this setting is the starting point to analyse structural transitions between icosahedral quasicrystals, following the methods proposed in Kramer (1987 ▶), Katz (1989 ▶) and Indelicato *et al.* (2012 ▶), which we are planning to address in a forthcoming publication.

These mathematical tools also have many applications in other areas. A prominent example is virology. Viruses package their genomic material into protein containers with regular structures that can be modelled *via* lattices and group theory. Structural transitions of these containers, which involve rearrangements of the protein lattices, are important in rendering certain classes of viruses infective. As shown in Indelicato *et al.* (2011 ▶), such structural transitions can be modelled using projections of six-dimensional icosahedral lattices and their symmetry properties. The results derived here therefore have a direct application to this scenario, and the information on the subgroup structure of the class of crystallographic representations of the icosahedral group and their intersections provides information on the symmetries of the capsid during the transition.

## Figures and Tables

**Figure 2:**
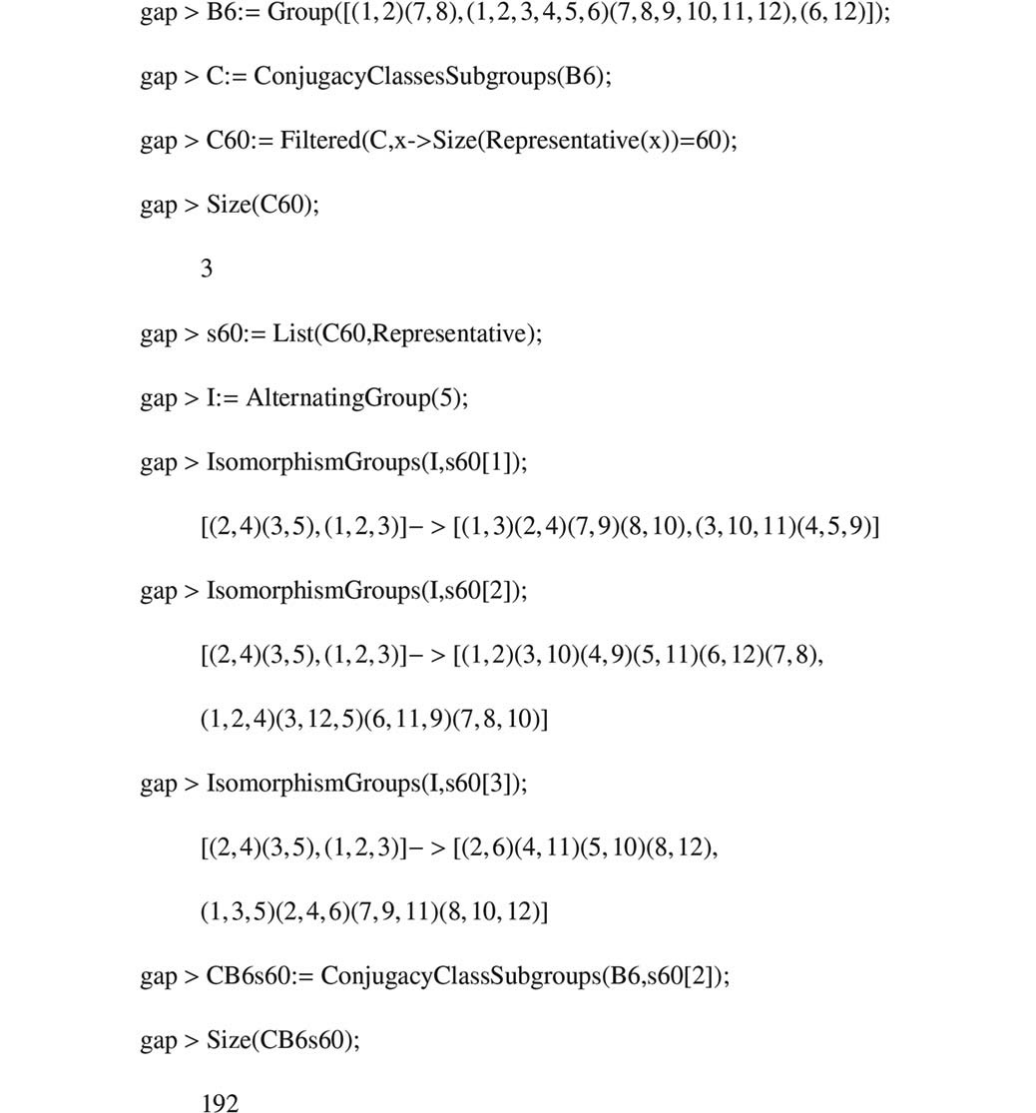
Algorithm 1.

**Figure 3:**
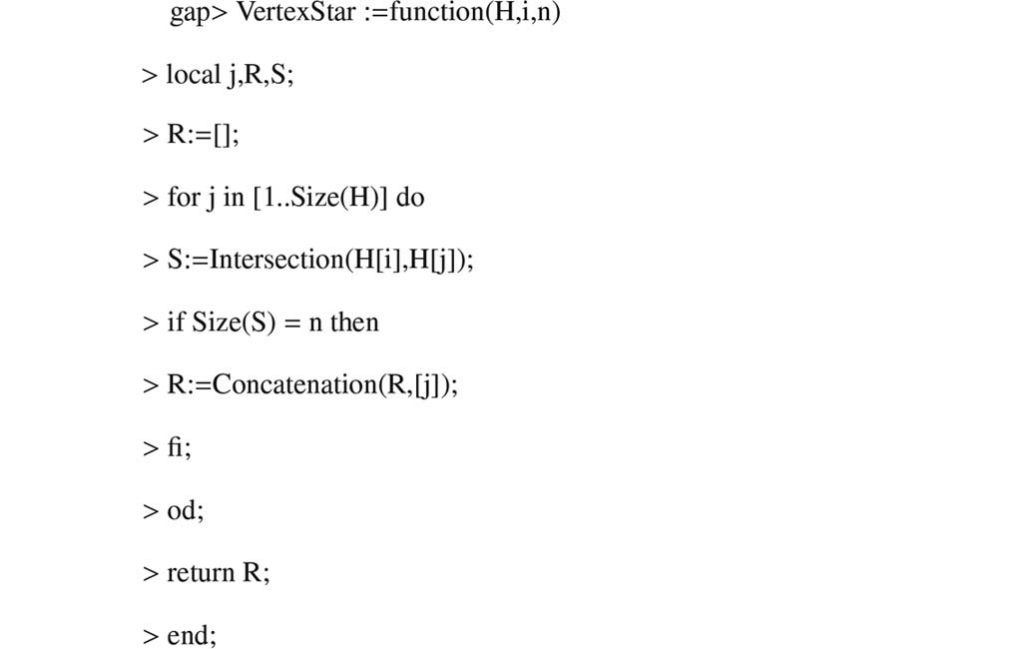
Algorithm 2.

**Figure 4:**
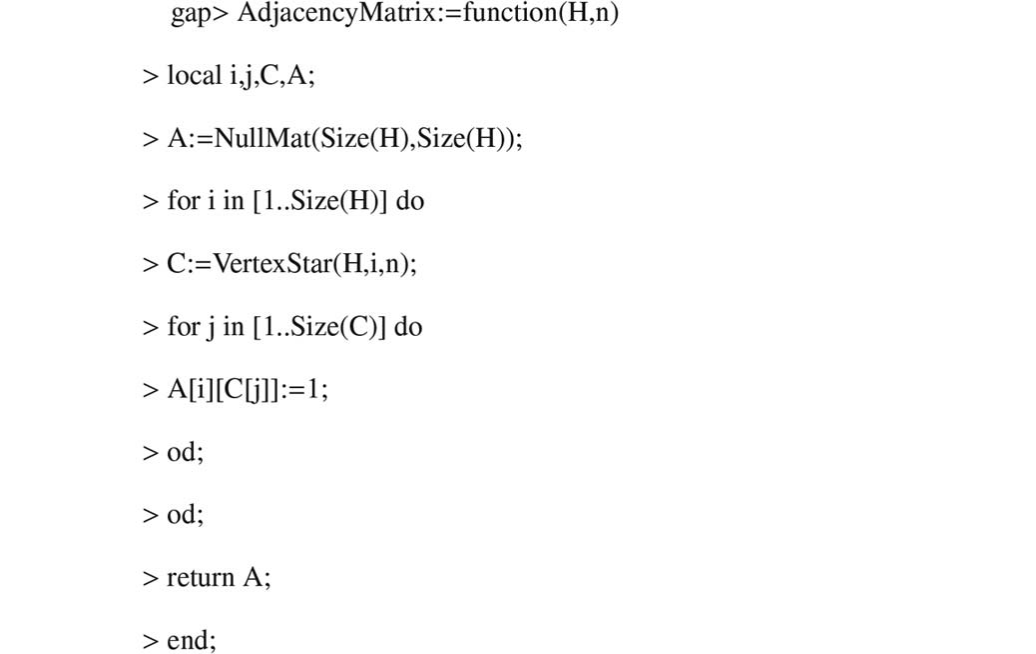
Algorithm 3.

**Figure 5:**
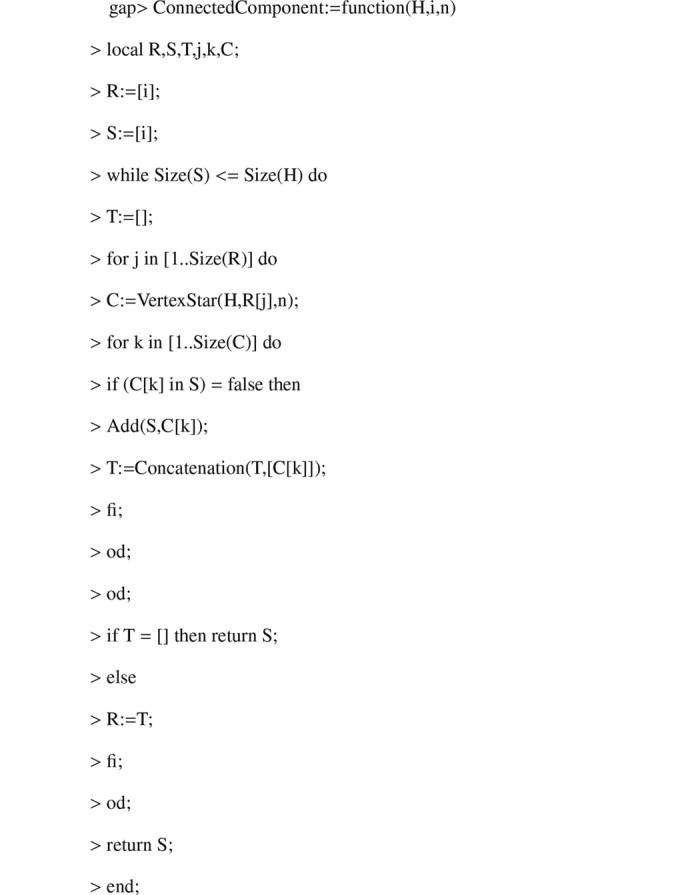
Algorithm 4.

**Figure 6:**
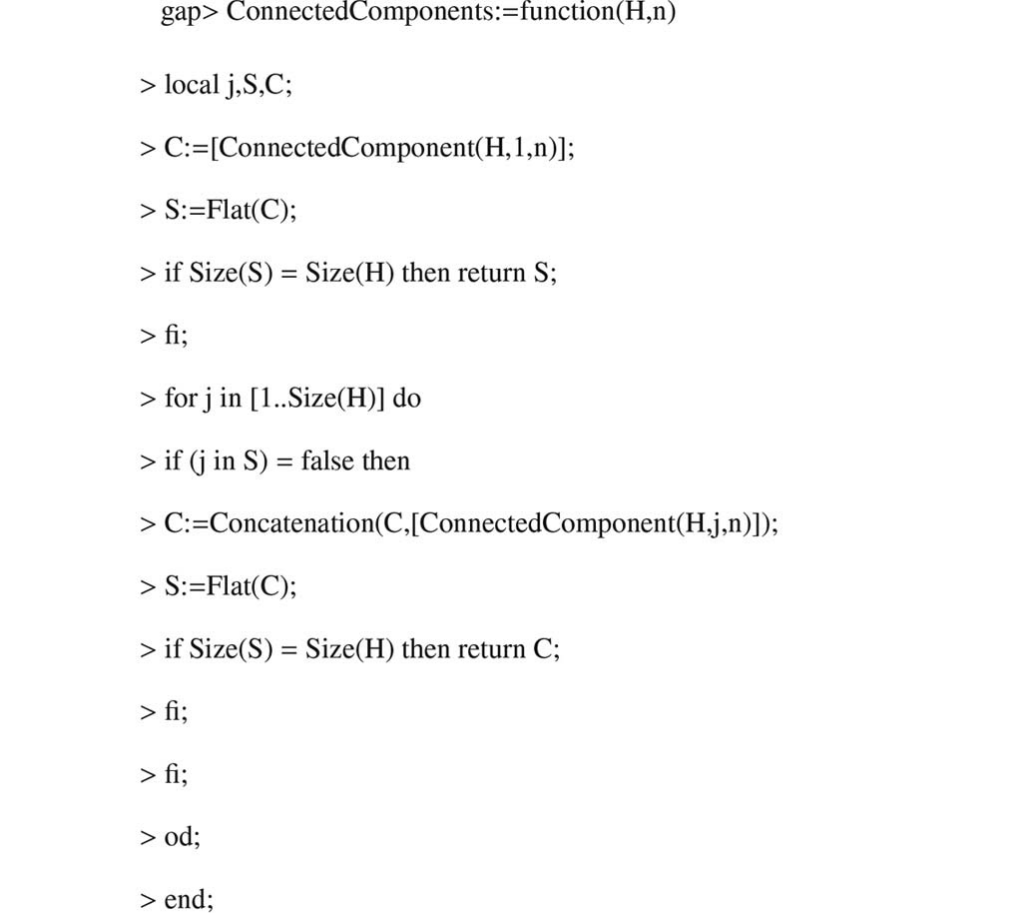
Algorithm 5.

**Table 1:** Character table of the icosahedral group Note that 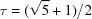 is the golden ratio.

	
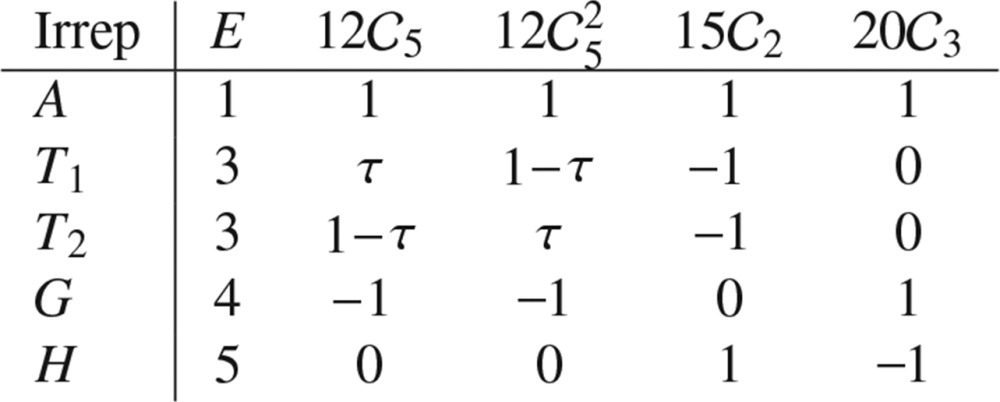	

**Table 2:** Explicit forms of the IRs 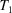 and 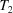 with 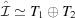

Generator	Irrep 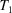	Irrep 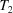
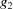	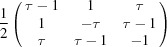	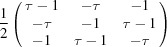
		
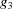	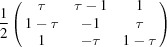	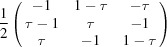

**Table 3:** Nontrivial subgroups of the icosahedral group 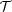
 stands for the tetrahedral group, 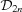 for the dihedral group of size 2*n*, and *C*_*n*_ for the cyclic group of size *n*.

Subgroup	Generators	Relations	Size
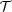	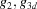	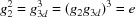	12
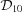	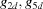	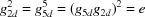	10
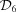	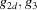	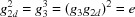	6
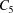	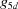	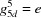	5
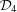	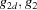	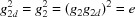	4
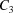	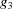	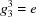	3
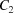	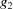	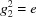	2

**Table 4:** Permutation representations of the generators of the subgroups of the icosahedral group

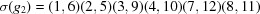
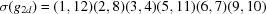
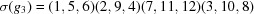
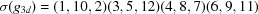
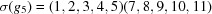
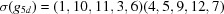

**Table 5:** Sizes of the classes of subgroups of the icosahedral group in 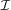 and *B*_6_

Subgroup	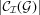	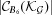
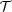	5	480
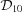	6	576
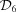	10	960
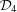	5	120
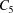	6	576
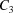	10	320
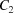	15	180

**Table:** The numbers highlighted are the indices of the graphs, and correspond to their degrees 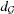.

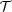 -graph		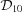 -graph		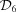 -graph		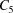 -graph	
Eig.	Mult.	Eig.	Mult.	Eig.	Mult.	Eig.	Mult.
**5**	1	**6**	6	**10**	6	**0**	192
3	45	2	90	2	90		
−3	45	−2	90	−2	90		
1	50	−6	6	−10	6		
−1	50						
−5	1						

**Table:** 

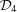 -graph		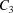 -graph		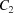 -graph		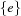 -graph	
Eig.	Mult.	Eig.	Mult.	Eig.	Mult.	Eig.	Mult.
**30**	1	**20**	2	**60**	2	**60**	1
18	5	4	90	4	90	12	5
12	5	−4	100	−4	90	4	90
6	15			−12	10	−4	90
2	45					−12	5
	31					−60	1
−2	30						
−4	45						
−8	15						

## Appendix A

In order to render this paper self-contained, we provide the character tables of the subgroups of the icosahedral group, following Artin (1991 ▶), Fulton & Harris (1991 ▶) and Jones (1990 ▶).

Tetrahedral group  []:

Dihedral group :

Dihedral group  (isomorphic to the symmetric group *S*_3_):

Cyclic group *C*_5_ []:

Dihedral group *D*_4_ (the Klein Four Group):

Cyclic group *C*_3_ []:

Cyclic group *C*_2_:

## Appendix B

Here we show the explicit forms of , the representations in  of the subgroups of , together with their decompositions in .

## Appendix C

In this Appendix[App appc] we show our algorithms, which have been implemented in *GAP* and used in various sections of the paper. We list them with a number from 1 to 5.

Algorithm 1 (Fig. 2 ▶): Classification of the crystallographic representations of  (see §4). The algorithm carries out steps 1–4 used to prove Proposition 4.1[Statement proposition4.1]. In the *GAP* computation, the class  is indicated as CB6s60. Its size is 192.

Algorithm 2 (Fig. 3 ▶): Computation of the vertex star of a given vertex *i* in the -graphs. In the following, *H* stands for the class  of the crystallographic representations of ,  denotes a vertex in the -graph corresponding to the representation  and *n* stands for the size of : we can use the size instead of the explicit form of the subgroup since, in the case of the icosahedral group, all the non isomorphic subgroups have different sizes.

Algorithm 3 (Fig. 4 ▶): Computation of the adjacency matrix of the -graph.

Algorithm 4 (Fig. 5 ▶): This algorithm carries out a breadth-first search strategy for the computation of the connected component of a given vertex *i* of the -graph.

Algorithm 5 (Fig. 6 ▶): Computation of all connected components of a -graph.
